# Preparation, Properties, and Applications of Graphene-Based Hydrogels

**DOI:** 10.3389/fchem.2018.00450

**Published:** 2018-10-01

**Authors:** Guochao Liao, Junfeng Hu, Zhou Chen, Ruiqian Zhang, Guanchun Wang, Tairong Kuang

**Affiliations:** ^1^Joint Laboratory for Translational Cancer Research of Chinese Medicine of the Ministry of Education of the People's Republic of China, International Institute for Translational Chinese Medicine, University of Chinese Medicine, Guangzhou, China; ^2^School of Mechanical and Power Engineering, Nanjing Tech University, Nanjing, China; ^3^Science and Technology on Reactor Fuel and Materials Laboratory, Nuclear Power Institute of China, Chengdu, China; ^4^Key Laboratory of Polymer Processing Engineering of Ministry of Education, South China University of Technology, Guangzhou, China

**Keywords:** graphene, graphene oxide, hydrogels, applications, preparation

## Abstract

As a new carbon-based nanomaterial, graphene has exhibited unique advantages in significantly improving the combination properties of traditional polymer hydrogels. The specific properties of graphene, such as high electrical conductivity, high thermal conductivity and excellent mechanical properties, have made graphene not only a gelator to self-assemble into the graphene-based hydrogels (GBH) with extraordinary electromechanical performance, but also a filler to blend with small molecules and macromolecules for the preparation of multifunctional GBH. It fully exploits the practical applications of traditional hydrogels. This review summarizes the preparation methods, properties, and the applications of GBH. Further developments and challenges of GBH are also prospected.

## Introduction

Graphene is a new nanomaterial with strict two-dimensional layers structure (Geim, 2009; Shi et al., 2018). With excellent mechanical, high electrical and thermal properties, graphene is the ideal filler for polymer-based nanocomposites (Li and Kaner, 2008). Hydrogel is the moderate crosslinked and branched polymer with three dimensional network structures (Yuk et al., 2017). It is widely studied and applied because of the ability to absorb large quantities of water, swell quickly, soft, elastine, and biologic compatibility (Smith et al., 2010; Qiu and Park, 2012). Graphene has exhibited unique advantages in significantly improving the combination properties of traditional polymer hydrogels (Xu et al., 2010a; Kostarelos and Novoselov, 2014). Graphene in hydrogels plays two roles: the gelator to self-assemble into the hydrogels, and the filler to blend with small molecules and macromolecules for the preparation of multifunctional hydrogels, which are collectively called graphene-based hydrogels (GBH) (Wang et al., 2016; Zhao et al., 2017).

This review aims to demystify the preparation methods, performance aspects, and the applications of GBH. Further developments and challenges of GBH are also prospected.

## Preparation methods and properties

### Self-assembly method

Self-assembly method means that the basic structure of graphene oxide (GO) is spontaneously translated into a stable 3D graphene structure under the interaction of non-covalent π-π bond. It is considered an effective approach for fabricating GBH. Various self-assembly methods such as hydrothermal process (Liu et al., 2017), chemical reduction (Sheng et al., 2011), and metal ion induced process (Cong et al., 2012) have been developed for 3D GBH. They can obtain some unique structures and characteristics, such as porous network structure, ultra-low density, excellent thermal properties, and thermal stability (Kuang et al., 2015).

Self-assembled GBH was first prepared by one-step hydrothermal method from GO solution in 2010 (Xu et al., 2010a). The moisture content of the GBH reached up to 97.4 wt%, and it demonstrated high levels of strength. Furthermore, the electrical conductivity of the GBH was 5 × 10^−3^ S/cm, and the storage modulus (450-490 kPa) was greater several orders than the traditional hydrogels. The 3D multifunctional GBH, which was self-assembled by combining DNA and GO sheets, possessed good mechanical property, big dye-adsorption capacity, and excellent self-cure capability (Xu et al., 2010b). In order to further improve the reduction degree of graphene for better GBH, some chemical reducing agent, such as hydrazine hydrate (Zhang and Shi, 2011), oxalic acid (Zhang et al., 2012), ascorbic acid (Shi et al., 2014) et al., were added to GO solution.

Compared with traditional hydrogel, an increase in strength and excellent energy storage performance has been found from self-assembled GBH. However, the applications of pure self-assembled GBH are restricted by their relatively low mechanical properties. Therefore, graphene used as high quality nano-fillers for polymer composite hydrogels is the focus of nowadays researches.

### Mixed solution method

It is a great challenge that single graphene sheets are easily caused agglomeration in the process of preparation and application, which leads to multilayer graphite. Compared with graphene, there are many oxygenous groups at the surface of GO, which can make GO perfect dispersion in polar solvents (water, ethanediol, DMF, and so on) (Paredes et al., 2008). The stable colloidal dispersion system usually occurs due to the strong hydrogen-bonds between GO and aqueous solution. In this case, mixed GO solution method is a very useful technology for fabricating GBH.

The graphene/gelatin hydrogel composite was fabricated by mixing graphene and gelatin solution (Tungkavet et al., 2015). The storage modulus response obviously enhanced with the graphene concentration increasing from 0 to 0.1%. The maximum of the highest storage modulus response and storage modulus sensitivity of graphene/gelatin hydrogel were 1.25 × 10^6^ Pa and 3.52, respectively. The sulfonated graphene (SG)/ poly(vinyl alcohol) (PVA) hydrogel, which was made by SG and PVA mixed solution, showed good mechanical property and intelligent adsorption property for cationic dyes compared to ordinary pure PVA hydrogel (Li et al., 2015). The tensile strength of the SG/PVA hydrogel increased with increasing SG, and the strength peak was as high as 37.34 kPa with 0.5wt% SG.

### *In-situ* polymerization

As a joint result of mixed GO, monomer-polymer, initiating agent, and other additives under certain conditions, *in-situ* polymerization of monomer-polymer occurs on the surface of GO, which leads to the final GO/polymer composite hydrogels. *In-situ* polymerization can be divided into two types based on the GO: the GO with and without treatment. The latter approach is grafting functional groups on the surface of GO, or stripping the nano-sheet layer from GO under ultrasonic and reacting with other monomers. GO also plays the role of cross-linking agent during the reaction. Such GO/polymer composite hydrogels possesses favorable dispersibility of GO, and uniform performance.

GO/polyacrylic acid (PAA) composite hydrogel was synthesized via cross-linking reaction of PAA at low temperature (Tai et al., 2013). The composite material displayed more excellent swelling characteristics and electrical response than the pure PAA hydrogel. GO/polyacrylamide (PAM) composite hydrogel was fabricated through *in situ* acrylonitrile polymerization in GO-water solution (Liu et al., 2012). The tensile strength of GO/PAM hydrogel was about 4.5 times higher than the pure PAM hydrogel, and the breaking elongation was 30 times exceeded than the PAM, but the content of GO was only 0.0079 wt%. GO/PAA hydrogel was prepared by *in situ* free radical polymerization triggered by graphene peroxide (Liu et al., 2013). It has the ability to self-heal when the fracture surfaces have maintained contact at low temperature or even room temperature for short periods. The recovery rate of the hydrogel can reach up to 88% at a prolonged healing time.

## Applications

**Figure 1:**
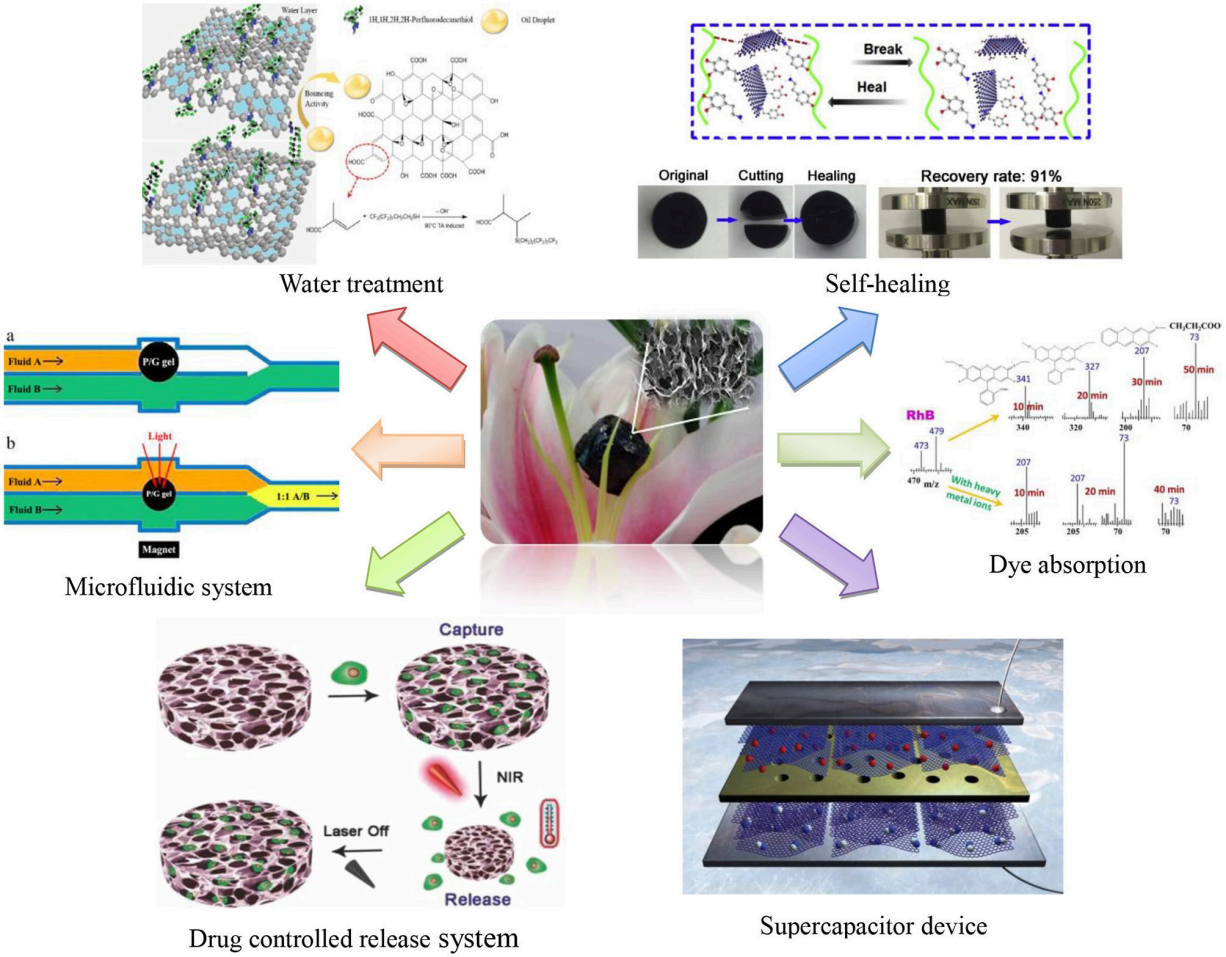
Applications of GBH.

The excellent performance of GBH is based on the inseparable synergy between hydrophobicity and π-conjugated structure in graphene sheets. GBH integrates mechanical strength, electrical conductivity, adsorption, hydroscopicity, water retention, controlled-release and biocompatibility together, and it will have broad application prospect in biomedical, supercapacitor, water treatment, dye absorption, catalyst carrier and intelligent response for microfluidic system (as shown in Figure 1).

### Biomaterials in biomedical field

The water-rich GBH is similar to natural soft tissues, in addition to the high conductivity, good mechanical strength, favorable biocompatibility, and the non-covalent bonds between graphene/GO and some polymer (chitosan (CS), poly(N,N-dimethylacrylamide) (PDMAA), etc.), GBH are attracted much attention in tissue engineering. Jing et al. studied the mussel-inspired GO/CS composite hydrogel, which was prepared by incorporating protein polydopamine (PDA) (Jing et al., 2017). As the GO concentration increases to a suitable quantity, the electrical conductivity of the hydrogel could reach up to 1.22 mS/cm. Meanwhile the strength was 3 times higher than pure CS hydrogel. More importantly, GO/CS hydrogel could improve cellular activities and proliferation of human enhancer of filamentation 1 (HEF1) and cardiomyocytes (CMs).

Meanwhile intelligent GBH prepared by graphene/GO and stimuli-responsive polymer (PAA, PVA, Poly(N-isopropy-lacrylamide) (pNIPAAm), etc.) has shown attractive prospects in drug controlled release system on account of the huge specific area of GO or graphene. The graphene/Ag (mass ratio of 1:5) composite hydrogel, which was made through the cross-linking reaction of graphene with acrylic acid and methylene bisacrylamide, exhibited good biocompatibility, and high swelling ratio (Fan et al., 2014). It displayed a significant acceleration of healing with the hydrogel in the treatment of artificial wounds in rats. More importantly, the composite hydrogel was used to facilitate complete reconstruction in 15-day wound-healing. Bai et al. prepared GO/PVA composite hydrogel and applied it in selective drug release under physiological pH (Bai et al., 2010). They found that 84% vitamin B_12_ molecules can be diffused from the hydrogel into neutral PBS solution (pH = 7.4) after 42 h. However, only 51% vitamin B_12_ molecules can be released in the acidic medium (pH = 1.7) at the same time. Li et al. successfully fabricated an NIR-responsive GBH as an active cell scaffold by pNIPAAm derivative solution and arginine-glycine-aspartic acid (RGD). The NIR-responsive GBH could efficiently capture cells, but also could release the cells on the stimulus of NIR light (Li et al., 2013).

### Supercapacitor

As the major electrochemical components for energy storage and release, supercapacitor should possess high specific capacitance, big reversible capacity, and long cycle life. GBH can be used as innovative electrode materials for supercapacitor, because of their unique surface structures and excellent conductive properties.

Tan et al. researched the viscoelastic properties and electrical conductivity of self-assembly GBH. They found that the apparent electrical conductivity of the GBH can be controlled in a range from 0.01 to 6 S/cm, which is sufficient for many electrical applications (Tan et al., 2014). Wang et al. prepared graphene/VO_2_ nanobelt composite hydrogel by using V_2_O_5_ and GO as precursors. They found that the graphene/VO_2_ hydrogel exhibited big specific capacitance (426 F/g), good cycling performance and high rate capability (Wang et al., 2013).

### Water treatment

The rapid development of petrochemical industry leads to a large number of industrial waste water, which contains significant amounts of acid, alkali salt, organic solvent, harmful dyes, or heavy metal ions. Hydrogel owns good hydrophilicity, at the same time it will not dissolve in water. The unique 3D crosslinked network structure makes hydrogel can be effective absorption and adsorption to high amounts of chemicals. The adsorptive abilities and adsorptive selectivity of hydrogel can be further improved by the huge specific surface area and electronegativity of graphene. Therefore hydrogel has shown a perfect foreground applied in sewage treatment and dye absorption.

LA/F/rGO hydrogel with good amphiphobicity was synthesized by hydrothermal process at low temperature with reduced GO, 1H,1H,2H,2H-perfluorodecanethiol (PF) and L-ascorbic acid (LA). The hydrogel possessed high selectivity to oil-water separating by pre-soaking (Hu et al., 2018). Dong et al. found that the Fe_3_O_4_/GO/polyacrylamide (PAM) hydrogel exhibited excellent mechanical property, high Photo-Fenton activity and big adsorption capacity. The hydrogel was achieved a 90% degradation of Rhodamine B (RhB, 20 mg/L) and a 72.7% degradation of chemical oxygen demand (COD, 2840 mg/L) in wastewater for 1 h under visible light (Dong et al., 2018). Organic pollutants and heavy metal ions could also be removed at the same time by the Fe_3_O_4_/GO/PAM hydrogel.

## Conclusion

The specific properties of graphene, such as high electrical conductivity, high thermal conductivity, and excellent mechanical properties, have made graphene not only a gelator to self-assemble into the GBH with extraordinary electromechanical performance, but also a filler to blend with small molecules and macromolecules for the preparation of multifunctional GBH. It fully exploits the practical applications of traditional hydrogels. In view of the developing trend of hydrogel in recent years, theoretical researches are relatively high. Researchers are very interested in the application prospect of hydrogel for biomedical, tissue engineering, supercapacitor, water treatment, dye absorption, catalyst carrier, and intelligent response for microfluidic system. However compared to the practical applications, the actual operation research is very weak. The formation mechanism of graphene/GO hydrogel in the aqueous solution and the influencing function are not apparent yet. Design and fabrication of GBH with new structures and functions will be a significant challenge in the future.

## Author contributions

All authors listed have made a substantial, direct and intellectual contribution to the work, and approved it for publication.

### Conflict of interest statement

The authors declare that the research was conducted in the absence of any commercial or financial relationships that could be construed as a potential conflict of interest.
